# Gibberellin–cytokinin regulation of compact plant architecture in *Atractylodes chinensis*

**DOI:** 10.7717/peerj.21597

**Published:** 2026-09-14

**Authors:** Zhijia Cao, Xiangyan Tu, Tongyan Zhu, Chaoyi Li, Chunying Zhao, Yang Lu

**Affiliations:** Hebei Province Key Laboratory of Study and Exploitation of Chinese Medicine, Chengde Medical University, Chengde, China

**Keywords:** *Atractylodes chinensis* (DC.) Koidz., Gibberellin, Cytokinin, Plant architecture, Multi-omics integration, Diterpenoid biosynthesis, Internode elongation, Molecular breeding

## Abstract

Research on high-quality *Atractylodes chinensis* (DC.) Koidz. germplasm is pivotal for its standardized cultivation, but the multi-omics mechanisms governing its plant architecture remain unclear. In this study, we integrated morphological quantification, RNA sequencing (RNA-seq), and liquid chromatography-tandem mass spectrometry (LC-MS/MS) to explore the molecular basis of architectural differences between a naturally compact line (JC) and the wild-type (WT). Morphological analysis confirmed that the JC line is characterized by significantly shorter internodes and increased branching compared to the WT. Transcriptomic analysis identified 7,271 differentially expressed genes (DEGs), specifically the downregulation of the gibberellin (GA) biosynthetic gene ent-kaurenoic acid oxidase (*KAO*) and the upregulation of the GA catabolic gene GA 2-oxidase (*GA2ox*) in the JC line. Parallel metabolomic profiling identified an accumulation of GA metabolic precursors (GA_9_, GA_19_, and GA_44_) and significantly higher cytokinin (CTK) content in JC relative to WT. These results suggest a GA-centric regulatory network where disrupted GA metabolism and enhanced CTK accumulation synergistically drive the compact architectural phenotype. This study elucidates the GA–CTK regulatory module as a core mechanism modulating internode elongation and branching, providing key candidate genes and foundational insights for the molecular breeding of elite *A. chinensis* germplasm.

## Introduction

*Atractylodes chinensis* (DC.) Koidz. is a perennial herb belonging to the Asteraceae family. According to Traditional Chinese medicine, its dried rhizome is widely used for its spleen-strengthening, dampness resolving, and wind-dispelling properties (China Medical Science Press, 2020). It has been extensively used in East Asia for the treatment of rheumatic disorders, digestive dysfunction, night blindness, and influenza (Zhang et al., 2025). In recent years, increasing market demand has led to a rapid decline in wild *A. chinensis* resources, making artificial cultivation the primary production mode. Plant architecture, which is determined by traits such as branch number, branch angle, and plant height, profoundly influences plant growth and yield (Huang et al., 2023). Branching, arising from axillary bud initiation and outgrowth, together with internode development, including internode length and number, collectively shapes plant architecture and directly affects cultivation density and yield potential (Hu, Sun & Wang, 2020; Yang et al., 2021). However, the endogenous regulatory mechanisms underlying morphological remodeling in *A. chinensis* remain poorly understood, thereby limiting the identification of elite germplasm and progress in molecular breeding.

Phytohormones are endogenous signaling molecules that regulate plant growth and development at extremely low concentrations, among which gibberellins (GA) and cytokinins (CTK) are key regulators of plant architecture. GA plays a crucial role in cell elongation and organ development, particularly in internode elongation and the suppression of axillary bud growth, which are major determinants of plant height, branching, and flowering (Xing et al., 2024; Griffiths et al., 2024; Stowe & Yamaki, 1957; Yan et al., 2022). At the molecular level, GA promotes plant elongation by regulating cellular expansion (Castro-Camba et al., 2022), and its role in plant architectural regulation is highly conserved across diverse plant species (He et al., 2024). In contrast, CTK exerts pleiotropic effects on plant growth and development, including the regulation of bud and root development, vascular differentiation, leaf senescence, and axillary bud activation (Zhou et al., 2024). Previous studies have demonstrated that CTK promotes the nuclear translocation of transcription factors, thereby stimulating cell division, a process essential for lateral shoot outgrowth (Yang et al., 2021). GA and CTK are considered core and conserved regulators of plant architecture in Asteraceae medicinal plants, including *A. chinensis*. By comparison, other phytohormones showed relatively weak or non-significant changes in our multi-omics analyses. Although the roles of GA and CTK in plant architectural regulation have been extensively studied, the multi-omics mechanisms underlying GA- and CTK-mediated morphological variation in *A. chinensis* remain largely unclear.

In this study, we selected two *A. chinensis* lines with distinct architectural phenotypes: the naturally compact line JC, characterized by dense branching and suitability for high-density cultivation, and the wild-type line WT, which exhibits fewer and more dispersed branches. Both lines were carefully screened and selected by our research team following 3 years of field observation. To investigate the hormonal and molecular basis underlying these morphological differences, we combined morphological trait quantification with metabolomic profiling using liquid chromatography–tandem mass spectrometry (LC–MS/MS) and transcriptomic analysis using RNA sequencing (RNA-seq) of young leaves. Specifically, this study aimed to characterize the major architectural differences between JC and WT, identify differentially expressed genes (DEGs) and differential metabolites (DMs) associated with morphological variation, and elucidate the roles of GA and CTK in regulating plant architecture in *A. chinensis*. Our findings provide new insights into the hormonal and molecular mechanisms underlying morphological regulation in *A. chinensis* and establish a foundation for future germplasm improvement and molecular breeding.

## Materials and Methods

### Plant materials and growth conditions

JC is a naturally mutated line of *A. chinensis* identified from a local wild population in Chengde, Hebei Province, China, after three consecutive years of field screening, whereas WT represents the original wild-type line. Both lines were taxonomically identified as *Atractylodes chinensis* (DC.) Koidz. by Professor Chunying Zhao of Chengde Medical University (Chengde, Hebei, China) based on morphological characteristics.

JC and WT plants were cultivated under identical environmental conditions, including light intensity, temperature, water supply, and soil conditions, and were sampled at the same developmental stage to minimize environmental and developmental influences on hormone responses. Morphological traits were measured using standardized criteria. Internode length was calculated as the mean length of 10 middle internodes per branch and recorded to the nearest 0.1 cm. Internode number was determined from two representative branches per plant, whereas branch number was defined as the total number of aboveground branches per plant. Morphological remodeling was defined as significant variation in internode length, internode number, and branch number between the two lines (Student’s *t*-test, *P* < 0.05). Three biological replicates were included for each line. For metabolomic and transcriptomic analyses, young leaves from JC and WT plants were collected and pooled in equal amounts from plants of the same line to reduce individual variation. All samples were immediately frozen in liquid nitrogen and stored at −80 °C until further analysis.

### Determination of endogenous hormones

#### Quantitative detection based on LC–MS/MS

Approximately 50 mg of finely ground plant tissue was weighed and extracted with one mL of methanol/water/formic acid solution (15:4:1, v/v/v) containing 10 μL of an internal standard mixture (100 ng/mL). After thorough mixing, internal standard normalization was applied to improve quantitative accuracy and minimize matrix interference. Samples were vortexed for 10 min and centrifuged at 12,000 rpm for 5 min at 4 °C. The supernatant was transferred to a new centrifuge tube and concentrated under reduced pressure. The dried extract was subsequently reconstituted in 100 μL of 80% methanol, filtered through a 0.22 μm membrane filter, and transferred to LC–MS/MS sample vials for analysis (Li et al., 2016; Floková et al., 2014).

Hormone metabolites were detected using ultra-high-performance liquid chromatography coupled with tandem mass spectrometry (UHPLC–MS/MS). Qualitative analysis was performed using the MWDB database (Metware Biotechnology Co., Ltd., Wuhan, Hubei, China), which was established based on authentic reference standards. Metabolite annotation confidence was classified into three levels: Level 1, identified using authentic standards; Level 2, putatively identified based on MS/MS spectra; and Level 3, predicted according to accurate mass information. All hormone metabolites identified in this study were classified as Level 1 metabolites. Quantitative analysis was conducted using the multiple reaction monitoring (MRM) mode of triple quadrupole mass spectrometry. Differential metabolites were screened based on fold change (FC) values, with metabolites showing FC ≥ 2 or FC ≤ 0.5 considered significantly different. The Kyoto Encyclopedia of Genes and Genomes (KEGG) database was used for metabolite annotation and metabolic pathway enrichment analysis.

#### Enzyme-linked immunosorbent assay

Young leaves were immediately snap-frozen in liquid nitrogen after sampling. Endogenous CTK contents were quantified using a plant-specific enzyme-linked immunosorbent assay (ELISA) kit (Cat. No. YJ020295; Mlbio, Shanghai, China) according to the manufacturer’s instructions. The assay included sample extraction, standard curve preparation, and absorbance measurement to ensure the accuracy and reliability of hormone quantification.

### RNA-seq analysis

Approximately 0.5 g of plant tissue from each sample was submitted to Majorbio Bio-Pharm Technology Co., Ltd. (Shanghai, China; https://www.majorbio.com) for RNA-seq library construction, with three biological replicates included for each line. Total RNA was extracted from the collected samples, and RNA concentration and purity were evaluated using a NanoDrop 2000 spectrophotometer (Thermo Fisher Scientific, Waltham, MA, USA). RNA integrity was assessed by agarose gel electrophoresis, and the RNA quality number (RQN) was determined using an Agilent 5300 system (Agilent Technologies, Santa Clara, CA, USA). Sequencing was performed on the NovaSeq X Plus platform (Illumina, San Diego, CA, USA) at Majorbio Bio-Pharm Technology Co., Ltd., Shanghai, China. Raw sequencing data were subjected to quality control and subsequent statistical analysis. *De novo* transcriptome assembly was conducted using Trinity (Grabherr et al., 2011). Clean reads from each sample were mapped to the Trinity-assembled reference transcriptome to generate alignment results for transcript quantification.

Differential expression analysis was performed using DESeq2 (Love, Huber & Anders, 2014), and genes with |log2FC| ≥ 1 and false discovery rate (FDR) < 0.05 were considered significantly differentially expressed. Benjamini–Hochberg correction was applied to control for multiple comparisons. Transcript abundance was quantified using RSEM (Li & Dewey, 2011) based on transcripts per million (TPM) values. TPM normalization was used to correct for differences in gene length and sequencing depth and is widely recognized as a standard method for transcriptome quantification.

The statistical power of the experimental design was calculated as 0.797 using the RNASeqPower tool. Three biological replicates are commonly accepted for omics studies of non-model medicinal plants. Quality control analysis of metabolite samples showed coefficients of variation (CV) <10%, indicating low experimental variation and high data reliability.

Functional enrichment analyses of DEGs included Gene Ontology (GO) and KEGG pathway analyses using GOATOOLS and the SciPy package in Python. GO terms and KEGG pathways with Bonferroni-corrected *P* < 0.05 were considered significantly enriched relative to the whole-transcriptome background. These statistical corrections were applied to improve the rigor and reliability of the enrichment analyses. Finally, a pathway diagram illustrating DEGs and DMs involved in diterpenoid biosynthesis was generated using BioGDP.com (Jiang et al., 2025).

### Statistical analysis

Statistical analyses were performed using Student’s t-test in SPSS version 25.0 (IBM Corp., Armonk, NY, USA). All data are presented as the mean ± standard deviation (SD). Differences were considered statistically significant at *P* < 0.05 and highly significant at *P* < 0.01. Statistical significance is indicated by asterisks in the figures and tables.

## Results

### Plant phenotype

**Figure 1:**
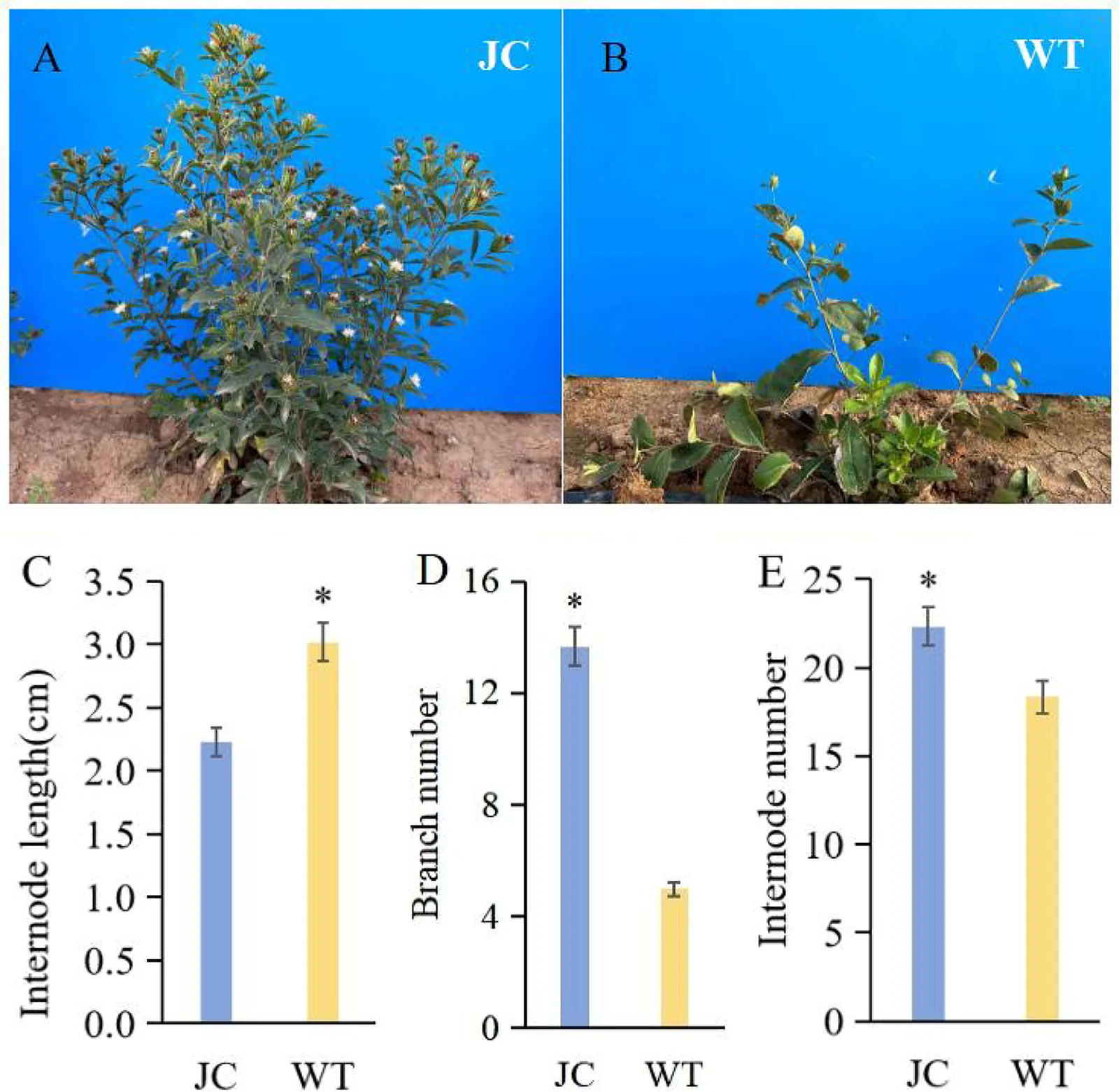
Analysis of plant morphology. (A) Whole-plant phenotypic characteristics of the naturally compact line JC; (B) whole-plant phenotypic characteristics of the wild-type (WT); (C–E) comparisons of internode length, branch number, and internode number between JC and WT, respectively. Data are presented as the mean ± SD from three biological replicates. Statistical significance was determined using Student’s t-test (*P < 0.05).

Distinct differences in plant architecture were observed between the JC and WT lines, indicating clear morphological remodeling characterized by significant variation in internode length, branch number, and internode number. JC exhibited a compact and erect growth habit (Fig. 1A), whereas WT displayed sparse branching and a broader canopy architecture (Fig. 1B). Quantitative analysis showed that the internode length of JC was 26.27% shorter than that of WT, representing a significant reduction (Fig. 1C). In contrast, JC produced substantially more branches and internodes than WT. The average branch number was 13.7 in JC compared with 5.0 in WT, while the average internode number was 22.3 and 18.3 in JC and WT, respectively, with both traits showing significant differences between the two lines (Figs. 1D and 1E).

### Analysis of differentially expressed genes

#### RNA-seq data quality control and *de novo* transcriptome assembly

To investigate the molecular basis underlying the phenotypic differences between the JC and WT lines, RNA-seq analysis was performed using the NovaSeq platform. After quality control of the raw sequencing data, 140,306,722 and 136,322,330 clean reads were obtained from the JC and WT groups, respectively. Both groups exhibited high sequencing quality, with per-base error rates below 0.02%, Q20 values exceeding 98%, Q30 values exceeding 95%, and GC contents greater than 45% (Table 1).

**Table 1:** Quality assessment of RNA-seq samples.

Sample	Raw reads	Raw bases	Clean reads	Clean bases	Error rate (%)	Q20 (%)	Q30 (%)	GC content (%)
JC_1	49,965,850	7,544,843,350	49,649,744	7,385,480,876	0.0122	98.66	95.75	45.52
JC_2	47,887,864	7,231,067,464	47,577,190	7,083,795,573	0.0121	98.67	95.81	45.45
JC_3	43,369,924	6,548,858,524	43,079,788	6,402,718,683	0.0122	98.63	95.68	45.43
WT_1	48,527,746	7,327,689,646	48,184,186	7,153,095,860	0.0121	98.7	95.91	45.7
WT_2	46,774,256	7,062,912,656	46,454,210	6,877,276,129	0.0121	98.67	95.8	46.03
WT_3	41,983,534	6,339,513,634	41,683,934	6,180,175,296	0.0121	98.67	95.81	45.74

**Note:**

Q20 and Q30 represent the percentages of bases with Phred quality scores ≥20 and ≥30, respectively. GC content indicates the percentage of guanine and cytosine bases in the clean reads.

*De novo* transcriptome assembly was performed using Trinity, and the clean reads from each sample were subsequently mapped to the assembled reference transcriptome. As shown in Table 2, the numbers of clean reads, mapped reads, and corresponding mapping rates were calculated for all biological replicates. All samples exhibited high mapping rates (≥84.38%), indicating that the assembled transcriptome accurately represented the transcriptional landscape of *A. chinensis*. These results demonstrate the high quality and reliability of the transcriptome dataset and provide a robust foundation for subsequent differential expression analysis.

**Table 2:** Read mapping statistics of JC and WT against the *de novo* assembled transcriptome.

Sample	Clean reads	Mapped reads	Mapped ratio (%)
JC_1	24,824,872	21,225,095	85.50
JC_2	23,788,595	20,159,258	84.74
JC_3	21,539,894	18,334,851	85.12
WT_1	24,092,093	20,525,269	85.20
WT_2	23,227,105	19,757,165	85.06
WT_3	20,841,967	17,719,713	85.02

**Note:**

Mapping rate (%) represents the proportion of clean reads successfully aligned to the *de novo* assembled transcriptome.

#### Identification and expression patterns of DEGs

**Figure 2:**
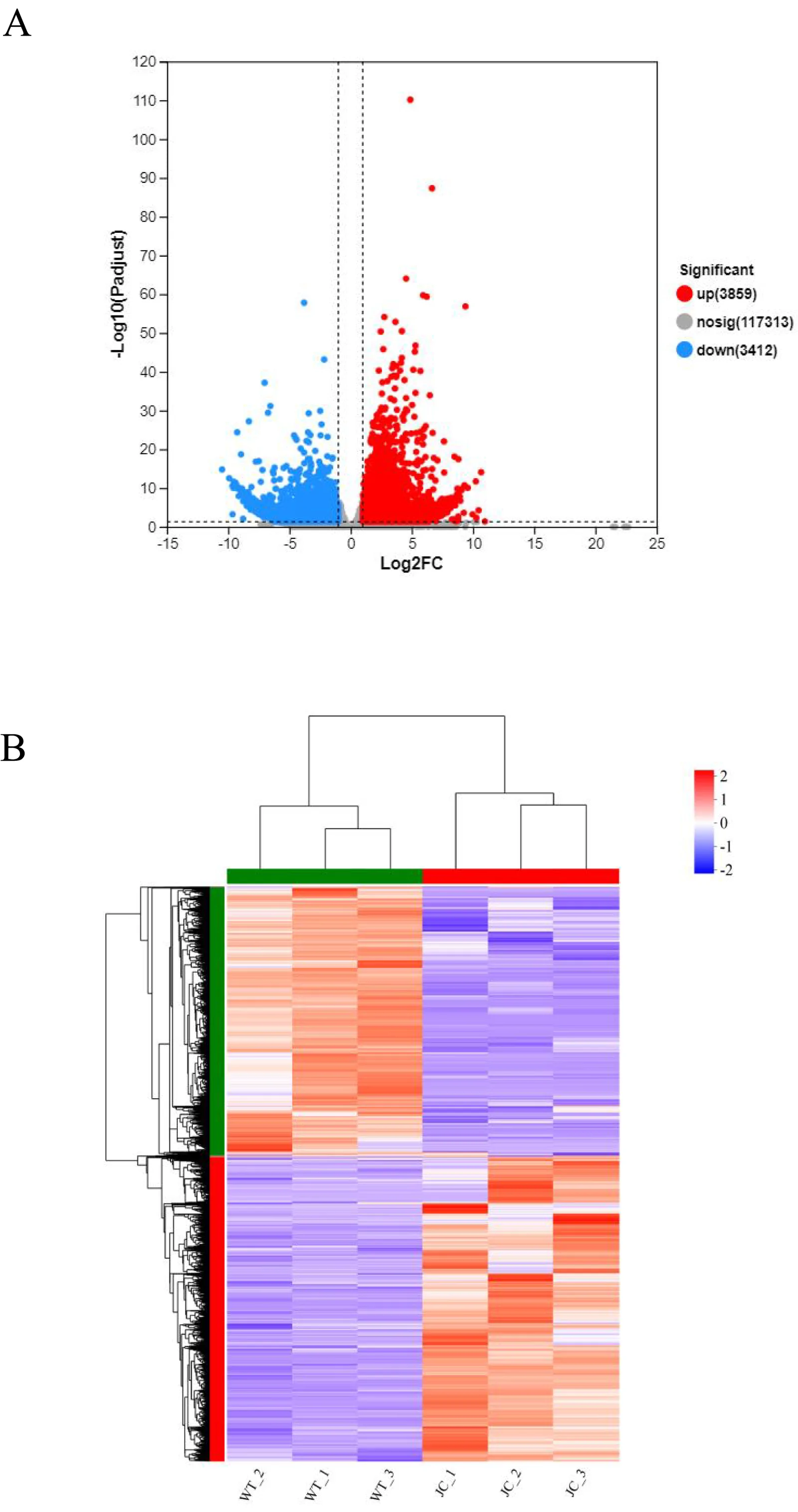
Analysis of differentially expressed genes (DEGs). (A) Volcano plot showing the distribution of upregulated and downregulated DEGs between JC and wild-type (WT); (B) hierarchical clustering heatmap showing the expression patterns of DEGs in JC and WT. Red indicates relatively high expression levels, whereas blue indicates relatively low expression levels.

Differential expression analysis identified 7,271 significantly DEGs in JC relative to WT, including 3,859 upregulated genes and 3,412 downregulated genes (Fig. 2A). The transcriptomic dataset demonstrated high biological reproducibility, stringent quality control, and reliable statistical support based on the screening criteria of |log2FC| ≥ 1 and FDR < 0.05, supporting the robustness and credibility of the DEG analysis. Hierarchical clustering analysis was further performed to visualize the expression patterns of the identified DEGs. In the heatmap, red indicates relatively high gene expression levels, whereas blue represents relatively low expression levels. The corresponding expression ranges are shown by the numerical scale adjacent to the color bar in the upper right corner of Fig. 2B.

#### Functional enrichment analysis of DEGs

**Figure 3:**
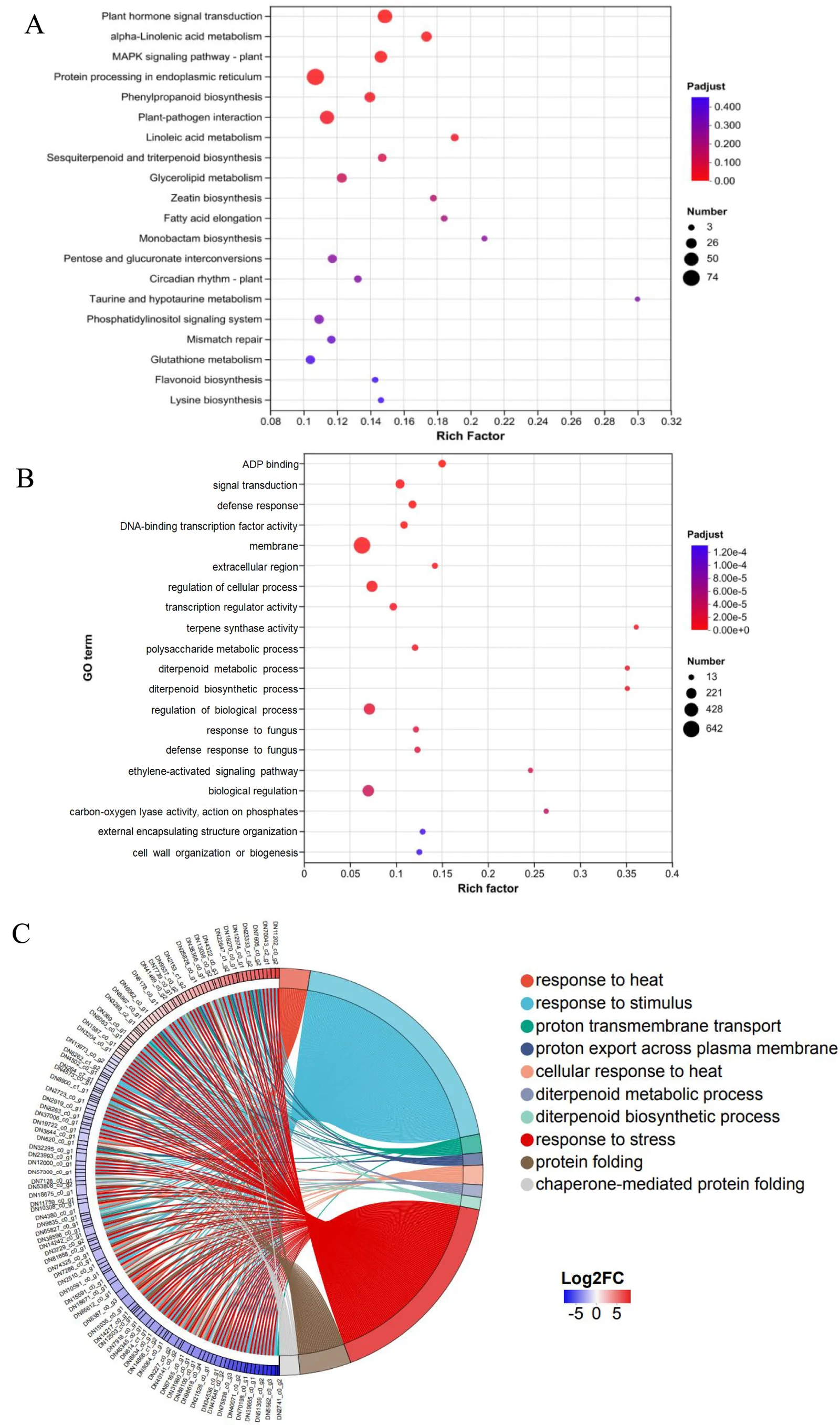
Functional enrichment analyses of differentially expressed genes (DEGs) between JC and wild-type (WT). (A) Kyoto encyclopedia of genes and genomes (KEGG) pathway enrichment analysis; (B) gene ontology (GO) enrichment analysis; (C) GO enrichment chord diagram showing the relationships between DEGs and significantly enriched GO terms.

To further explore the biological functions of the identified DEGs, KEGG pathway enrichment analysis was conducted for the JC and WT comparison. A total of 1,365 DEGs were significantly enriched in 129 pathways. As shown in Fig. 3A, the most significantly enriched pathways included plant hormone signal transduction (map04075), alpha-linolenic acid metabolism (map00592), MAPK signaling pathway–plant (map04016), protein processing in the endoplasmic reticulum (map04141), phenylpropanoid biosynthesis (map00940), and plant–pathogen interaction (map04626). Specifically, two pathways closely associated with hormone regulation, namely plant hormone signal transduction and zeatin biosynthesis, were significantly enriched, suggesting potential involvement of GA and CTK in architectural regulation.

In parallel, GO enrichment analysis showed that the DEGs were significantly enriched in two GA-related biological processes: diterpenoid metabolic process (GO:0016101) and diterpenoid biosynthetic process (GO:0016102) (Fig. 3B). The GO enrichment chord diagram further illustrates the relationships between DEGs and enriched GO terms (Fig. 3C). These enrichment analyses suggest that GA- and CTK-related pathways play important roles in regulating plant architecture in *A. chinensis*.

### Analysis of hormones by LC–MS/MS and ELISA

To validate the transcriptomic findings related to hormone-mediated morphological remodeling, targeted metabolomic profiling was performed in both the JC and WT lines. Based on the predefined screening criteria, a total of 15 DMs were identified.

Among these, three auxin-related metabolites were detected: N-(3-indolylacetyl)-L-leucine (IAA-Leu), 3-indoleacetonitrile (IAN), and tryptamine (TRA). TRA was significantly enriched in the tryptophan metabolism (ko00380) and indole alkaloid biosynthesis (ko00901) pathways. Three CTK-related metabolites were also identified, including N6-isopentenyladenosine-5′-monophosphate (iPRMP), 2-methylthio-N6-isopentenyladenosine (2MeSiPR), and kinetin (K). Compared with WT, iPRMP was significantly downregulated in JC, whereas 2MeSiPR and K were significantly upregulated.

In addition, three GA metabolic precursors, namely GA_9_, GA_19_, and GA_44_, showed significant accumulation in JC relative to WT. Among them, GA_9_ and GA_19_ were significantly enriched in the diterpenoid biosynthesis pathway (ko00904). Five jasmonic acid-related metabolites were identified, including OPC-4, H2JA, OPDA, and JA-ILE. JA, OPC-4, H2JA, and JA-ILE were upregulated in JC, whereas OPDA was downregulated. JA was significantly enriched in alpha-linolenic acid metabolism (ko00592) and plant hormone signal transduction (ko04075), while JA-ILE was specifically enriched in the plant hormone signal transduction pathway (ko04075). Furthermore, the strigolactone metabolite 5-deoxystrigol (5DS) was significantly downregulated in JC and enriched in carotenoid biosynthesis (ko00906).

**Figure 4:**
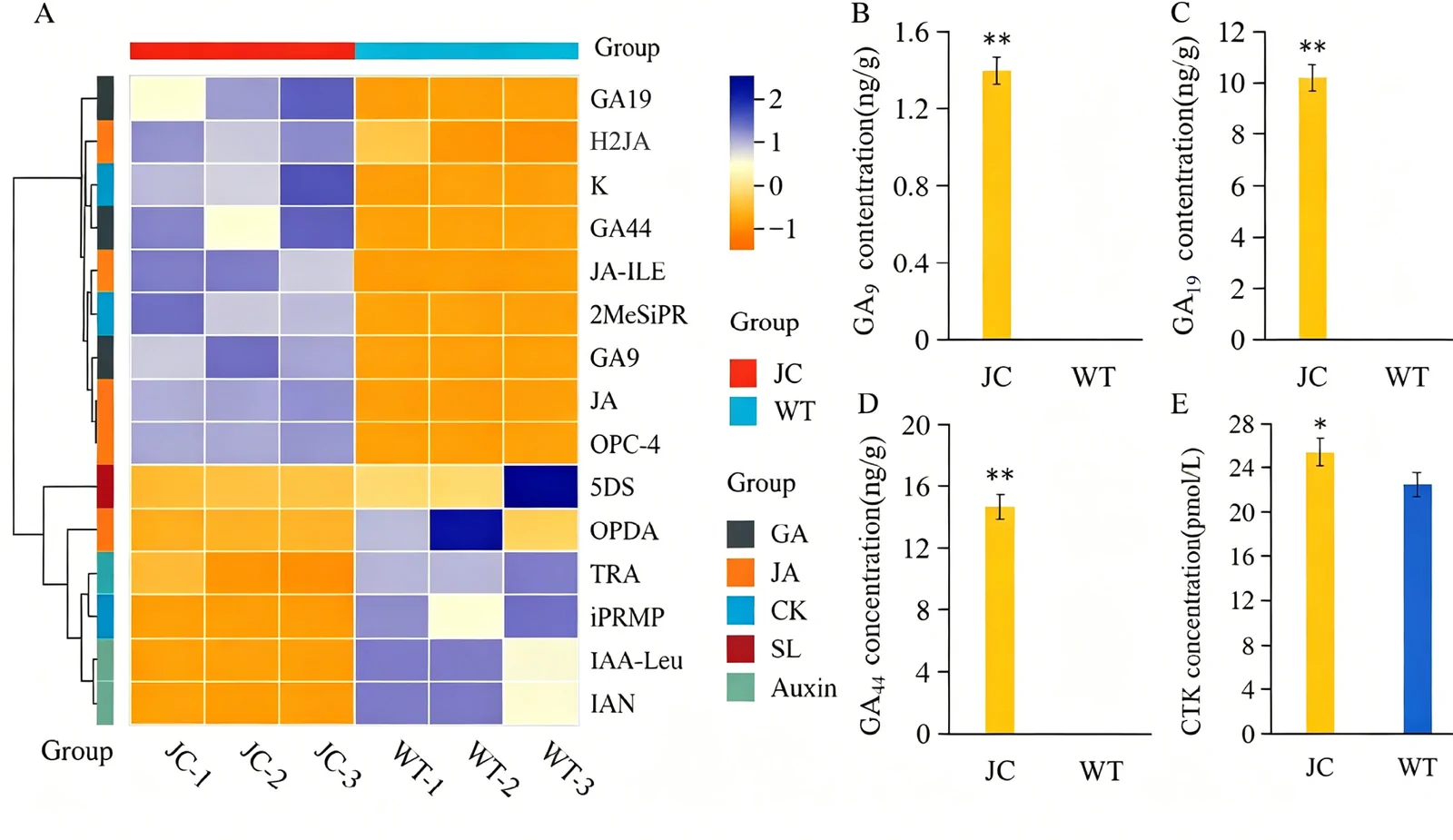
Plant hormone metabolic analysis between JC and wild-type (WT). (A) Hierarchical clustering heatmap of differential metabolites (DMs) involved in plant hormone metabolism. Blue represents high metabolite content and yellow represents low content; (B–E) Contents of GA_9_, GA_19_, GA_44_ (ng/g), and cytokinin (CTK) (pmol/L) in JC and WT. Data are presented as the mean ± SD from three biological replicates. Statistical significance was determined using Student’s *t*-test (*P < 0.05, **P < 0.01).

The clustering heatmap of DMs clearly separated JC from WT, indicating distinct metabolite accumulation patterns between the two lines. In the heatmap, blue indicates relatively high metabolite abundance, whereas yellow indicates relatively low metabolite abundance (Fig. 4A).

Because transcriptomic analysis revealed significant enrichment of GA- and CTK-related pathways, we further quantified GA and CTK contents. Consistent with the transcriptomic results, metabolomic analysis confirmed that the levels of GA metabolic precursors, including GA_9_, GA_19_, and GA_44_, were significantly higher in JC than in WT (Figs. 4B–4D). In addition, ELISA-based quantification demonstrated that CTK content was significantly elevated in JC relative to WT (Fig. 4E).

### DEGs and DMs related to diterpenoid biosynthesis

**Figure 5:**
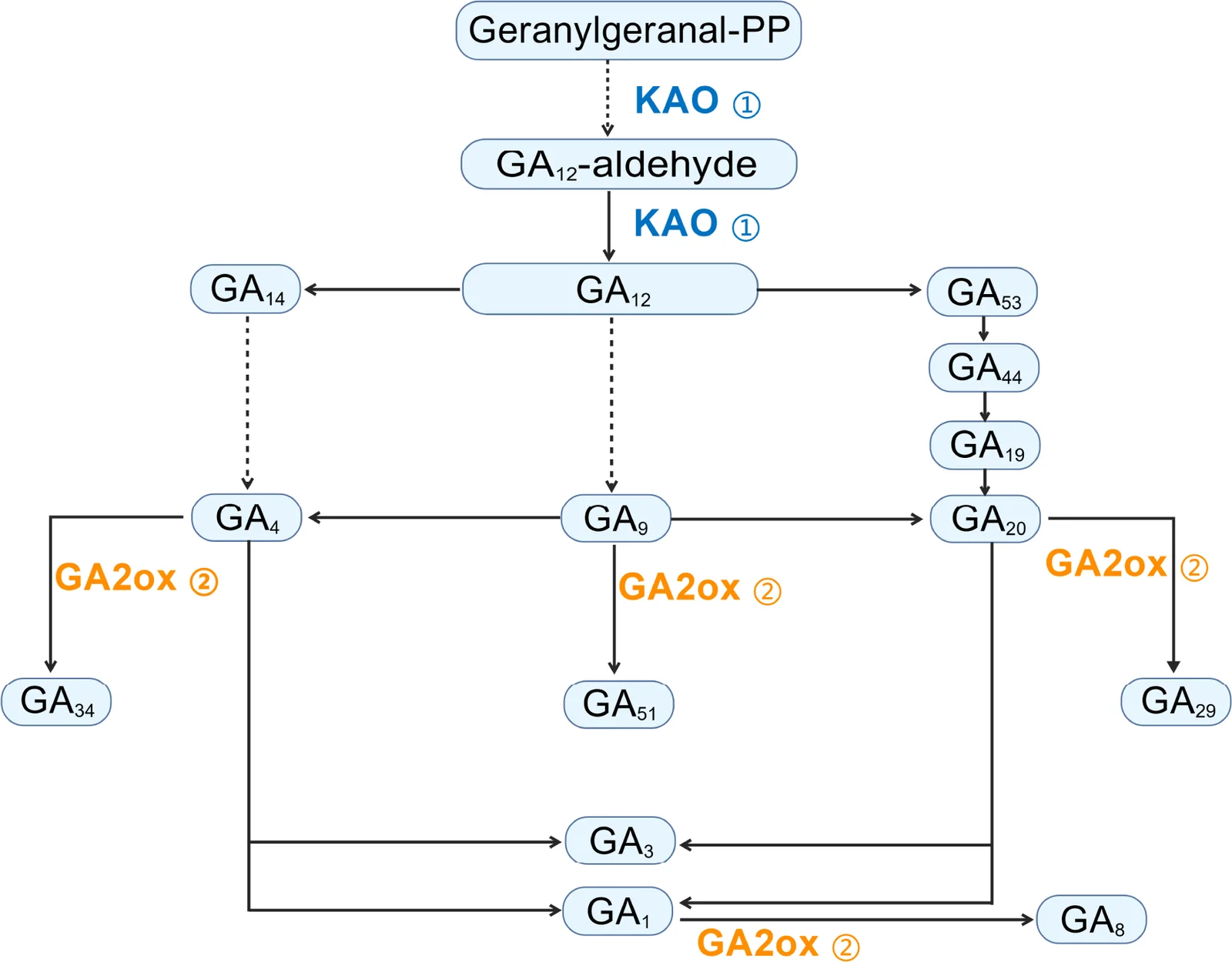
Integrated analysis of differentially expressed genes (DEGs) and differential metabolites (DMs) involved in the diterpenoid biosynthesis pathway between JC and wild-type (WT). The pathway diagram illustrates the coordinated regulation of gibberellin metabolism and cytokinin accumulation associated with plant architectural variation in *A. chinensis*. ①: ent-kaurenoic acid oxidase (KAO); ②: GA 2-oxidase (GA2ox).

To further investigate the molecular basis underlying architectural variation in *A. chinensis*, the diterpenoid biosynthesis pathway associated with GA metabolism was reconstructed through integrated analysis of DEGs and DMs (Fig. 5). The reconstructed pathway highlights the coordinated regulation of GA metabolism and CTK accumulation, including the downregulation of ent-kaurenoic acid oxidase (KAO), upregulation of GA2-oxidase (GA2ox), accumulation of GA metabolic precursors, and elevated CTK content, all of which are associated with internode elongation and plant architectural regulation.

Two key DEGs were mapped to the diterpenoid biosynthesis pathway: *TRINITY_DN32144_c0_g1*, encoding KAO, and *TRINITY_DN59398_c0_g1*, encoding GA2ox. Compared with WT, *TRINITY_DN32144_c0_g1* was significantly downregulated in JC, whereas *TRINITY_DN59398_c0_g1* was significantly upregulated. Concurrently, the GA metabolic precursors GA_9_, GA_19_, and GA_44_ accumulated significantly in JC. These results suggest that coordinated alterations in DEGs and DMs associated with the diterpenoid biosynthesis pathway play important roles in the morphological regulation of *A. chinensis*.

## Discussion

This study elucidated the hormonal basis underlying morphological remodeling in *A. chinensis* through integrated analyses of transcriptomic, metabolomic, and morphological data. Compared with WT, the JC line exhibited significantly shorter internodes but markedly increased branch and internode numbers, resulting in a more compact plant architecture. The greater number of branches and internodes in JC may contribute to an increased photosynthetically active leaf area and enhanced nutrient assimilation capacity under the same cultivation density. The present study primarily focused on primary metabolic processes associated with GA and CTK biosynthesis and catabolism, which are directly involved in the regulation of plant architecture. In contrast, secondary metabolic pathways related to medicinal terpenoid biosynthesis were beyond the scope of this work and will be investigated in future studies. Because plant architecture is regulated by multiple endogenous and environmental factors, phytohormones are considered central regulators of this process. Through integrated transcriptomic and metabolomic analyses of the JC and WT lines, we identified a series of DEGs and DMs associated with architectural variation and further characterized the key metabolic pathways potentially involved in the morphological regulation of *A. chinensis*.

In plants, GA and CTK are key regulators of morphological development and play central roles in shaping plant architecture. GA primarily promotes internode elongation and stem growth, leading to increases in plant height and internode length (Wu et al., 2023). Previous studies in *Arabidopsis* demonstrated that GA suppresses axillary bud formation by modulating the activity of the DELLA–SPL9 complex, thereby inhibiting shoot branching (Zhang et al., 2020). In contrast, CTK promotes branching by activating axillary buds and stimulating lateral shoot outgrowth (Waldie & Leyser, 2018). The interaction between GA and CTK is largely antagonistic, and the balance between their accumulation and signaling activities finely regulates plant architectural traits. Such hormonal crosstalk is essential for coordinating plant growth and developmental plasticity in response to changing environmental conditions.

Bioinformatic analyses confirmed the differential expression of *KAO* and *GA2ox* in the JC line, providing important molecular evidence for their potential involvement in the regulation of compact plant architecture. As a member of the CYP88A subfamily of cytochrome P450 monooxygenases, KAO catalyzes the conversion of ent-kaurenoic acid (KA) to GA_12_, a common precursor of bioactive GAs, and therefore plays a pivotal role in regulating endogenous GA biosynthesis (Regnault et al., 2014). In contrast, the *GA2ox* gene family belongs to the 2OG-Fe(II) oxygenase superfamily and is characterized by the conserved 2OG-Fe(II)_Oxy domain. *GA2ox* genes function primarily in GA deactivation through 2β-hydroxylation, which represents one of the major pathways of GA catabolism (Zhu et al., 2023). Early studies identified GA2ox enzymes that specifically target C19-GAs, including bioactive GAs and their direct precursors such as GA_9_ and GA_20_, which are classified as class I and II 2ODD oxidases (Yamaguchi, 2008; Li et al., 2014; Thomas, Phillips & Hedden, 1999). Subsequently, additional GA2ox enzymes acting on C20-GAs were identified and classified as class III 2ODD oxidases (Lee & Zeevaart, 2005). These enzymes reduce the accumulation of bioactive GAs by consuming precursor molecules such as GA_12_ and GA_53_ (Regnault et al., 2014). Although more than 136 GAs have been identified in plants, fungi, and bacteria, only a limited number, including GA_1_, GA_3_, GA_4_, GA_5_, GA_6_ and GA_7_, are considered biologically active (Salazar-Cerezo et al., 2018; Zhang, 2011). Moreover, GA and CTK exhibit antagonistic regulatory effects in meristem activity and plant developmental processes (Wu et al., 2016).

In the present study, transcriptomic analysis revealed significant downregulation of the GA biosynthetic gene *KAO* and upregulation of the GA catabolic gene *GA2ox* in JC. Consistently, metabolomic analysis demonstrated significant accumulation of the GA metabolic precursors GA_9_, GA_19_, and GA_44_ in the JC line. These findings suggest that altered GA metabolism in JC may reduce the conversion efficiency of GA precursors into bioactive GAs, thereby restricting internode elongation and contributing to the compact architectural phenotype. The markedly shorter internodes observed in JC relative to WT are likely associated with the coordinated downregulation of *KAO* and upregulation of *GA2ox*. In addition, CTK content was significantly higher in JC than in WT. Given the antagonistic relationship between GA and CTK in the regulation of internode elongation and shoot branching, the coordinated modulation of these two hormones may play a central role in shaping the compact architecture of JC. These results indicate that the morphological variation observed in *A. chinensis* is closely associated with altered GA metabolism and elevated CTK accumulation. Although the GA–CTK regulatory module appears to be highly conserved among Asteraceae species, the downstream regulatory networks associated with rhizome development and medicinal component accumulation may represent species-specific characteristics in *A. chinensis*.

Through integrated transcriptomic, metabolomic, and phytohormone quantification analyses, this study provides insights into the molecular mechanisms underlying morphological development in *A. chinensis*. The results suggest that GA promotes internode elongation by enhancing cell expansion, whereas CTK positively regulates shoot branching. The significant enrichment of DEGs in hormone-related signaling pathways further highlights the important role of phytohormonal regulation in shaping plant architecture. In addition to GA and CTK, significant differences were also observed in other hormones, including IAA and JA, between the JC and WT lines, suggesting that multiple hormone signaling pathways may participate in the regulation of morphological variation in *A. chinensis*. However, the precise regulatory functions of these hormones require further investigation.

These findings provide valuable molecular resources for future breeding programs targeting desirable architectural traits and contribute to a broader understanding of the molecular basis of growth and development in *A. chinensis*. Nevertheless, the present study was based on a static comparison between the JC and WT lines and did not include time-course analyses or functional validation experiments. Future studies should incorporate dynamic hormone profiling, rhizome tissue analyses, exogenous hormone treatments, and gene functional verification to further clarify the causal relationships between GA/CTK signaling and morphological development. In addition, because KEGG and GO databases are primarily derived from model plant species, the species-specific regulatory pathways of *A. chinensis* require further validation through improved genome annotation and functional genomic analyses. These efforts will provide a stronger theoretical and molecular foundation for germplasm improvement and molecular breeding in *A. chinensis*.

## Conclusion

This study investigated the molecular basis of morphological variation in *A. chinensis* through integrated transcriptomic, metabolomic, phytohormone, and morphological analyses. Compared with the WT line, the compact JC line exhibited shorter internodes and increased branching, reflecting distinct architectural characteristics. Multi-omics analyses identified significant alterations in GA- and CTK-related pathways, including downregulation of the GA biosynthetic gene KAO, upregulation of the GA catabolic gene GA2ox, accumulation of GA metabolic precursors, and elevated CTK content in the JC line. These coordinated changes were associated with reduced internode elongation and enhanced branching, suggesting that altered GA metabolism and CTK accumulation may contribute to compact plant architecture in *A. chinensis*. In addition, key DEGs and DMs identified in this study provide candidate molecular markers and potential targets for future germplasm improvement. The findings expand current understanding of hormone-associated architectural regulation in *A. chinensis* and provide a foundation for further investigation of the underlying regulatory networks. However, because the present study was based on comparative multi-omics analyses without functional validation, future research should incorporate time-course analyses, exogenous hormone treatments, and gene functional studies to verify the proposed roles of GA–CTK interactions in morphological development.

## Supplemental Information

10.7717/peerj.21597/supp-1 Supplemental Information 1 raw data.

## Additional Information and Declarations

### Data Availability

The following information was supplied regarding data availability:

The data can be accessed from The China National GeneBank Sequence Archive (CNSA) with the project accession number CNP0008428.
